# In Vitro Inhibition of Neutrophil Elastase Activity by Inhaled Anti-Pseudomonas Antibiotics Used in Cystic Fibrosis Patients

**DOI:** 10.1155/2010/809591

**Published:** 2010-06-16

**Authors:** Andreas Hector, Matthias Kappler, Matthias Griese

**Affiliations:** Pediatric Pulmonology, Children's Hospital, University of Munich, 80337 Munich, Germany

## Abstract

*Background*. Inhaled antibiotics are commonly used in the treatment of cystic fibrosis lung disease. A previous study suggested neutrophil elastase activation by colistin in vitro. Here, we investigated direct effects of the commonly used antibiotics colistin and tobramycin on neutrophil elastase activity. *Methods*. Neutrophil elastase was measured spectrophotometrically. The antibiotics colistin and tobramycin were added in different concentrations with or without the addition of albumin. *Results*. Generally, neutrophil elastase activity was lower in the absence of albumin compared to its presence. Both antibiotics, colistin and tobramycin, had inhibitory effects on neutrophil elastase activity except for high concentrations of colistin when albumin was absent. *Conclusions*. Our results suggest inhibitory effects of colistin and tobramycin in vitro. There was a clear dependency of neutrophil elastase measurements on the presence of albumin. Clinical studies are needed to investigate potential direct effects of inhaled antibiotics on neutrophil elastase activity in cystic fibrosis airways.

## 1. Background

Cystic fibrosis is the most common recessive disease in Caucasians with a prevalence of about 1 : 2500 [1]. Cystic fibrosis is characterized by progressive lung destruction due to chronic neutrophilic airway inflammation and bacterial infection [2]. Most of the morbidity and mortality of cystic fibrosis patients results from the lung disease [3]. Large amounts of neutrophils are targeted into the airways, mostly by the chemokine interleukin 8, where they are primed, activated and engage bacterial phagocytosis releasing high amounts of oxidants and proteases [4]. Neutrophils derived from cystic fibrosis patients release significantly higher levels of neutrophil elastase when activated by interleukin 8 compared to healthy subjects or bronchiectatic patients [5]. It was suggested that neutrophils in cystic fibrosis airway environment undergo necrosis, rather than apoptosis [6, 7]. This leads to uncontrolled secretion of interleukin 8 with further chemotaxis and accumulation of neutrophils and an overwhelming release of neutrophil elastase. Collateral damage of airway surface epithelium and destruction of neighboring structures allow bacterial persistence in niches and further perpetuation of inflammation [8, 9]. With ongoing cystic fibrosis lung disease, a state termed as prolonged endobronchial protease activity (PEPA) is established [10]. We recently found that phagocytic activity is reduced in cystic fibrosis neutrophils supposedly due to proteolytic cleavage of the specific interleukin 8 receptor CXCR1 which plays a pivotal role in neutrophil phagocytosis of bacteria most likely by neutrophil elastase [11].

Since the introduction of antibiotics in the mid 1950s and the development of new antibiotic regimens, the mean survival in cystic fibrosis patients dramatically increased [12]. After intravenous or inhaled antibiotic therapy bacterial burden is reduced in the airways of cystic fibrosis patients, as is neutrophil burden, free elastase, and associated inflammatory response [13–16]. Inhaled treatment of cystic fibrosis lung disease commonly includes nebulized antibiotics such as colistin and tobramycin [17]. In addition to indirect mechanisms of the antibiotics to reduce host inflammation by the killing of bacteria and reduced concomitant inflammation, direct actions of the antibiotics on the proteolytic milieu are possible. Indeed, a previous study suggested neutrophil elastase activation by colistin in cystic fibrosis patients in vitro [18]. The purpose of this study was to compare directly, side by side, the effects of colistin and tobramycin on neutrophil elastase activity.

## 2. Methods

### 2.1. Neutrophil Elastase Spectrophotometric Assay

Neutrophil elastase activity was measured with the specific peptide substrate methoxysuccinyl-ala-ala-pro-val-p-nitroanilide (MSAAPV-pNA—Sigma-Aldrich, Munich, Germany) as previously described by Hilliard et al. [19]. Purified human neutrophil elastase (Elastin Products Company, Owensville, MO, USA) was diluted to final concentrations of 0.5, 1, 2, 4, and 8 *μ*g/mL in an assay buffer (Dulbecco's phosphate buffered saline, PAA Laboratories, Pasching, Austria) in the presence or absence of albumin (0.1% bovine serum albumin, Fermentas, St. Leon-Rot, Germany) to test whether the presence of additional protein has an effect on neutrophil elastase measurements. To investigate effects of antibiotics on neutrophil elastase measurements, colistin (colistimethate sodium salt, Colistin CF, Gruenenthal, Aachen, Germany) and tobramycin (Tobi, Novartis, Nuernberg, Germany) at concentrations of 1, 10, 100 and 1000 *μ*mol/L were added to neutrophil elastase for 10 min (20°C) prior to analysis. As a control for colistin we used the vehicle buffer provided by the manufacturer to dissolve the antibiotics. As a control for tobramycin we dissolved 11.25 mg of sodium chloride in 5 mL aqua ad iniectabilia and adjusted the pH to 6.0 according to the product sheet of the manufacturer. Directly after addition of the substrate, 96-well plates (Greiner Bio-One, Frickenhausen, Germany) were measured at 405 nm absorbance on a plate reader (Anthos, Krefeld, Germany). Samples were measured in duplicates and a standard curve (range 0.5–8 *μ*g/mL) was included within the assay.

The used substrate is highly sensitive and specific to neutrophil elastase [20, 21], and change of optical density (OD) is directly proportional to the rate of p-nitroanilide cleavage; thus change of OD indicates a change in specific activity [19]. Neutrophil elastase activity was calculated from the slope of the over-time increase in color formation (change of OD) at a wavelength of 405 nm. The slopes were calculated by linear regression analysis of each experiment.

### 2.2. Effect of Antibiotics and Albumin on Neutrophil Elastase Activity

To test effects of antibiotics on neutrophil elastase measurements, colistin and tobramycin were added in different concentrations (final concentration of 1, 10, 100, 1000 *μ*M) to neutrophil elastase in presence or absence of albumin. Following a preincubation of 10 minutes the substrate MSAAPV-pNA was added directly before measurements and results were compared to samples without antibiotics or albumin. The minimal inhibitory concentration for tobramycin is 8 *μ*g/mL [16], whereas the antibiotic breakpoint for colistin is 2 *μ*g/ml [22]. Since inhaled antibiotics are known to reach high levels in airways, we used concentration ranges from 1 to 1000 *μ*M for tobramycin and colistin, which is equal to 0.47 to 467 *μ*g/mL and 1.16 to 1155 *μ*g/mL, respectively.

### 2.3. Statistics

Data are presented as mean or mean +/− standard error of mean for the number of independent experiments indicated. The nonparametric Wilcoxon signed rank test was performed for comparison of neutrophil elastase measurements in the presence of albumin compared to that in the absence of albumin. Repeated-measures analysis of variance with Bonferroni post-hoc test was conducted to analyze the effect of different concentrations of antibiotics on neutrophil elastase measurements in the presence or absence of albumin. Differences were considered statistically significant if *P* values were *P* < .05.

## 3. Results

**Figure 1:**
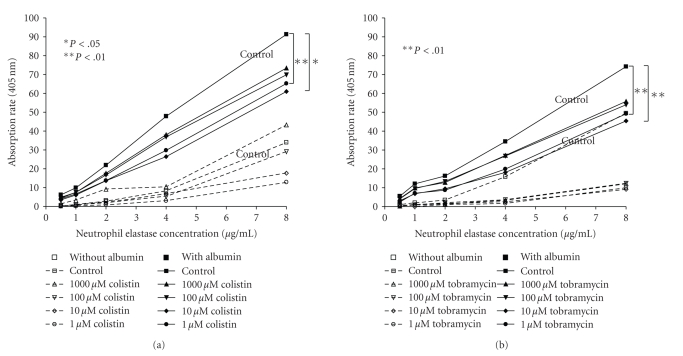
Effects of colistin (a) and tobramycin (b) on neutrophil elastase measurement (absorption) are given for different concentrations of colistin and tobramycin (1, 10, 100, 1000 *μ*M) either in presence (filled symbols, continuous lines) or in absence (blank symbols, dashed lines) of albumin. Data are presented as means of *n* = 3. *P < .05; **P < .01 compared to controls.

P-nitroanilide cleavage by neutrophil elastase was concentration-dependent over the range of neutrophil elastase investigated (Figure 1) and was linear for various time periods up to 30 minutes (not shown).

To test the effects of inhaled antibiotics used in the treatment of cystic fibrosis patients on neutrophil elastase measurements in vitro, we added colistin and tobramycin to the assay. The addition of either colistin or tobramycin led to reduced substrate cleavage. This was significant for 1 *μ*M and 10 *μ*M of colistin (*P* < .01 and *P* < .05, resp.) and for 1 *μ*M and 10 *μ*M of tobramycin (*P* < .01). At very high concentrations no or only very small effects were noted (Figure 1).

**Figure 2:**
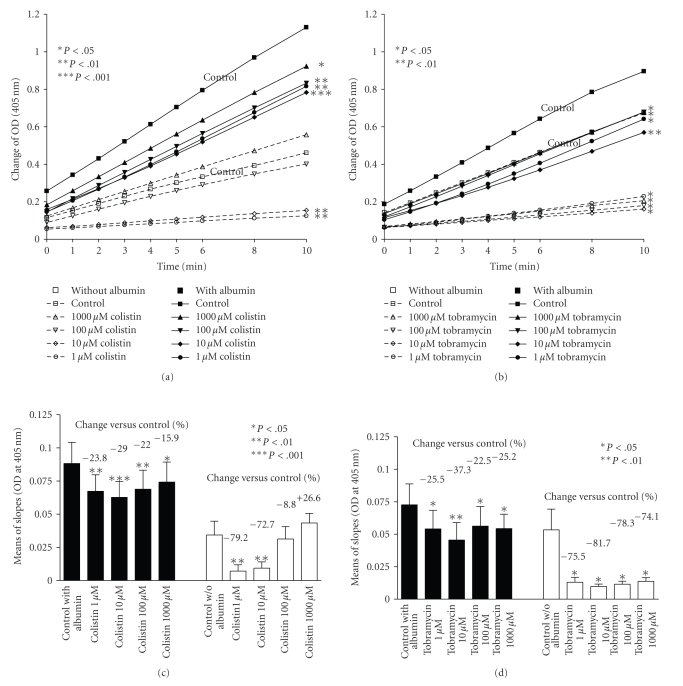
Effect of colistin (a) and (c) and tobramycin (b) and (d) in different concentrations (1, 10, 100, 1000 *μ*M) on neutrophil elastase activity measurements (neutrophil elastase concentration = 8 *μ*g/mL) in presence (filled symbols and contineous lines or filled columns) or absence (blank symbols and dashed lines or empty columns) of albumin. In Figures 2(a) and 2(b) change of optical density (OD) over time is given (mean of *n* = 3), in Figures 2(c) and 2(d) mean values for slopes of changes in OD over 10 minutes are expressed as means and SEM (bars) and % changes compared to respective control are given of *n* = 3. *P < .05; **P < .01; ***P < .001 compared to controls.

To assess if the presence of proteins could affect neutrophil elastase measurements, we added bovine serum albumin. p-nitroanalide cleavage was generally higher in the presence of albumin than in its absence (*P* < .0001). In order to assess neutrophil elastase activity, the change of optical density over time was calculated from the slope of the time curves (Figure 2) and the slopes were compared to each other.

Lastly, we investigated the effects of the antibiotics either in presence or absence of albumin. In the presence of albumin, colistin significantly inhibited neutrophil elastase activity by 16% to 24% (*P* < .01 for 1 *μ*M and 100 *μ*M; *P* < .001 for 10 *μ*M; *P* < .05 for 1000 *μ*M) and tobramycin by 23% to 37% (*P* < .05 for 1 *μ*M, 100 *μ*M and 1000 *μ*M; *P* < .01 for 10 *μ*M) compared to controls without antibiotics. Without albumin, colistin significantly inhibited neutrophil elastase by 73% to 79% at 1 *μ*M and 10 *μ*M (*P* < .01) and tobramycin by 74 to 82% (*P* < .05) (Figure 2). 

Similar effects were observed for the other neutrophil elastase concentrations (data not shown). 

## 4. Discussion

Colistin and tobramycin are widely used nebulized antibiotics in cystic fibrosis patients and their efficacy in treatment of *P. aeruginosa* and the clinical amelioration of cystic fibrosis patients was shown in many studies [14–17, 23–25]. Surprisingly, a previous study suggested an activating effect of colistin on neutrophil elastase activity in vitro [18].

Therefore, we investigated direct effects of the antibiotics colistin and tobramycin used in patients with cystic fibrosis for inhalation on the measurements of neutrophil elastase activity. Neutrophil elastase activity was inhibited by both antibiotics except for high colistin concentrations. There was no significant enhancing effect of these antibiotics, both in the presence and absence of albumin. However, neutrophil elastase activity was also dependent on albumin and was significantly elevated in its presence.

These in vitro data suggest that both colistin and tobramycin may be slightly inhibitory on neutrophil elastase activity. This result is in contrast to the previous study by Jones et al. [18] in which the effect of colistin on neutrophil elastase activity in cystic fibrosis sputum samples at increasing colistin concentrations (3.9–500 *μ*M) was studied and neutrophil elastase activity was already increased at lowest colistin levels (3.9 *μ*M) compared to control incubations without the addition of colistin.

Reasons for these different results may include different colistin preparations and assay conditions used. As colistin represents mainly polymyxin E, however, more than 30 minor components have been isolated [26] and its behavior is critically dependent on its source and preparation which were not indicated in the previous study. The presence of protein significantly affects activity; however this does not change the direction of the modulatory effect of the antibiotics, whereas its magnitude was influenced.

On the other hand, our results might lead to the assumption that albumin itself had activating effects on neutrophil elastase and reduced inhibitory effects of the antibiotics on neutrophil elastase. This seems to be unlikely, because there was no dose-dependent change of neutrophil elastase activity by the antibiotics. If protein-protein interactions between albumin and the antibiotics affect the pharmacokinetic activity of neutrophil elastase, higher concentrations of antibiotics would have had an influence on the measurements. Instead, we suppose that the strong dependency of neutrophil elastase activity on albumin was due to variable binding of neutrophil elastase or substrate to tube surfaces, modulating the availability of these components to neutrophil elastase. The assay in the study of Jones et al. [18] was performed without addition of proteins like albumin. But the presence of proteins in the assay may more likely represent the real situation in cystic fibrosis airways where high concentrations of protein are present [27], affecting neutrophil elastase activity and therefore the experimental set-up with the presence of protein may more likely represent the conditions in cystic fibrosis airways. Whereas our results suggest that both tobramycin and colistin, have no activating effect on neutrophil elastase, it is obvious that one cannot predict in vivo effects from these data. Therefore, clinical studies must assess potential direct effects of inhaled antibiotics on neutrophil elastase activity in cystic fibrosis airways.
